# β-Caryophyllene Reduces the Inflammatory Phenotype of Periodontal Cells by Targeting CB2 Receptors

**DOI:** 10.3390/biomedicines8060164

**Published:** 2020-06-17

**Authors:** Giacomo Picciolo, Giovanni Pallio, Domenica Altavilla, Mario Vaccaro, Giacomo Oteri, Natasha Irrera, Francesco Squadrito

**Affiliations:** 1 Department of Biomedical, Dental, Morphological and Functional Imaging Sciences, University of Messina, Via C. Valeria, 98125 Messina, Italy; giacomopicciolo94@gmail.com (G.P.); daltavilla@unime.it (D.A.); oterig@unime.it (G.O.); 2 Department of Clinical and Experimental Medicine, University of Messina, Via C. Valeria, 98125 Messina, Italy; gpallio@unime.it (G.P.); vaccaro@unime.it (M.V.); nirrera@unime.it (N.I.); 3 SunNutraPharma, Academic Spin-Off Company of the University of Messina, Via C. Valeria, 98125 Messina, Italy

**Keywords:** β-Caryophyllene, CB2 receptors, inflammation, oral mucositis, periodontitis

## Abstract

Human gingival fibroblasts (GF) and human oral mucosa epithelial cells (EC) with an inflammatory phenotype represent a valuable experimental paradigm to explore the curative activity of agents to be used in oral mucositis. The role of cannabinoid receptor 2 (CB2) has not yet been investigated in oral mucositis. The aim of this study was to evaluate the therapeutic potential of β-Caryophyllene (BCP), a CB2 agonist, in an in vitro model of oral mucositis. GF and EC were stimulated with LPS (2 µg/mL) alone or in combination with BCP; a group of LPS challenged GF and EC were treated with BCP and AM630, a CB2 antagonist. LPS increased the inflammatory cytokines TNF-α, IL-1β, IL-6 and IL-17A whereas it decreased the anti-inflammatory cytokine IL-13. The upstream signals were identified in an augmented expression of NF-κB and STAT-3 and in reduced mRNA levels of PPARγ and PGC-1α. BCP blunted the LPS-induced inflammatory phenotype and this effect was reverted by the CB2 antagonist AM630. These results suggest that CB2 receptors are an interesting target to develop innovative strategies for oral mucositis and point out that BCP exerts a marked curative effect in a preclinical model of oral mucositis which deserves to be confirmed in a clinical setting.

## 1. Introduction

Oral mucositis (OM) is a clinical condition characterized by a marked inflammatory reaction that results in erythematous lesions, ulcers, dysphagia and inability to afford and ensure a physiological calories intake that ultimately leads to interrupting life-saving treatments in cancer patients [1,2,3,4]. Oral mucositis may also complicate and/or be a clinical manifestation of peri-implantitis a common complication of dental implants, showing a prevalence of at least 20% in patients that have undergone this surgical procedure [5,6,7,8]. As a direct consequence of this tremendous impact on public health, there is an urgent need to identify the exact physiopathology of this condition in order to facilitate the design of rational therapeutic strategies to cure this complication.

Indeed, the mechanism underlying oral mucositis has been, at least in part, clarified. Whatever the triggering event, either DNA damage as in the case of chemotherapy/radiation therapy or infectious stimuli and progressive bone loss as in peri-implantitis [9,10], a common pathway converges in an exaggerated production of reactive oxygen species (ROS) released by both epithelial cells and gingival fibroblasts [11]. In fact, ROS boosts the translocation to the nucleus of Nuclear Factor Kappa B (NF-κB) [12]. Once it reaches the nucleus, the transcription factor turns on genes that codify for inflammatory cytokines such as Tumor Necrosis Factor (TNF-α), Interleukin 1 beta (IL-1β) and IL-6; moreover at the same time, it silences genes priming anti-inflammatory signals, in particular IL-13 and the nuclear receptor called peroxisome proliferator-activated receptor gamma (PPAR-γ) [13,14]. Indeed, this NF-κB primed cytokine storm may be considered an important arm of the acute phase response, but it has also been reported to have a key role in driving the second wave of the acute response in mammals. This second step of the inflammatory cascade is a crucial phenomenon that, at least at the beginning, is positive and reinforces the host immune and inflammatory response. The master regulator of this second step of host response to inflammatory injury is the Signal Transducer and Activator of Transcription (STAT) 3 [12]. STAT3 belongs to the seven-member family of proteins that cause the transduction of hormonal information from the cell membrane to the nucleus. STAT3 primes the formation of T helper 2 (Th2) cells that release a large number of Th2 derived cytokines including IL-17A. Activation of STAT3 is induced by several hormones, being the best studied and analyzed the components of the IL-6 group of cytokines. More specifically IL-6 has been shown to cause a robust activation of STAT3, representing this molecular event either the triggering of the second phase and the pathophysiological link between the first wave and the second wave of the host immune-inflammatory reaction. However, if the second wave of the inflammatory response is not appropriately modulated, a transition may occur into a maladaptive response that transforms acute inflammation into a chronic inflammatory condition that is responsible for several disabling diseases.

Therefore, pharmacological modulation of either the first phase or the second phase of the host inflammatory response is a rational strategy for the treatment of oral mucositis.

Relevant bioassays are therefore needed to facilitate the preclinical screening of candidate molecules. In this context, human gingival fibroblasts (GF) and human oral mucosa epithelial cells (EC) with an inflammatory phenotype represent a valuable experimental paradigm to explore the potential curative activity of agents to be used in this clinical condition [15]. LPS boosts an inflammatory cascade that plays a pivotal role in the pathogenesis of this unpleasant disease. Indeed, LPS belongs to the pathogen associated molecular patterns (PAMPs) family, a group of molecules that orchestrate an exaggerated immune-inflammatory response. Therefore, LPS stimulation of epithelial cells and gingival fibroblasts is an appropriate model to study in vitro oral mucositis.

The endocannabinoid system has two classical receptors: the cannabinoid receptor of type 1 (CB1) and the cannabinoid receptor of type 2 (CB2). While the CB1 mediates the classical psychotropic effects, the CB2 is expressed in the immune system and exerts anti-inflammatory effects [16]. The endocannabinoid system, through the CB2 receptor, has a fundamental role in the modulation of the inflammatory signals during pathological conditions such as osteoarthritis and rheumatoid arthritis. CB2 receptor activation inhibits upstream and downstream molecules of the inflammatory process. In addition, stimulation of the CB2 receptors exerts analgesic activity that might be of clinical relevance in the management of patients suffering from oral mucositis. All these experimental evidences clearly suggest that the type 2 cannabinoid receptor is strategical for a rational innovative drug design. However, no study so far has investigated the hypothesis of targeting the CB2 receptor to modulate the inflammatory cascade that occurs in oral mucositis.

β-caryophyllene (BCP) is a Food Drug Administration (FDA) approved natural compound that engages cannabinoid CB2 receptors and causes anti-inflammatory and analgesic effects [17]. The aim of this study was to evaluate the therapeutic potential of BCP in an “in vitro” experimental paradigm of oral mucositis.

## 2. Materials and Methods

### 2.1. Cell Cultures

Human primary gingival fibroblasts (atcc-pcs-pcs201-018) and human oral mucosa epithelial cells (cticc1.8.3 sk0251) were obtained from LGC Standards S.r.l Milan, Italy and Clinisciences s.r.l. Rome, Italy, respectively. Cells were put in culture in a medium made by DMEM, 10% fetal calf serum, 1% antibiotic mixture and incubated at 37 °C with 5% of CO_2_. AM630 was put in the cell culture 2 h before BCP. Cells and cell supernatants were collected following an incubation of 4 h with all the substances.

### 2.2. Treatments of Cells

GF and EC cells were cultured in six well culture plates at a density of 2.5 × 10^5^ cells/well and were challenged with LPS (2 μg/mL; Escherichia coli serotype 055:B5; Sigma-Aldric, Milan, Italy) alone or with BCP (Sanherb Biotech Inc., China) at the dose of 10 μg/mL. A previous study showed that this dose represents the IC50 of the biomolecule at least in the experimental paradigm of LPS stimulated human chondrocytes and considering IL-1β as readout of this bioassay [18]. Furthermore, a set of LPS challenged GF and EC cells were treated with BCP (10 μg/mL) and AM630 (100 nM; Sigma-Aldric, Milan, Italy), an antagonist of the CB2 receptor. AM630 was added 2 h before BCP treatment. Cells were harvested after a 4 h of incubation with several treatments.

### 2.3. MTT Assay

Cell viability was evaluated by MTT assay. GF and EC cells were grown and then treated with LPS (2 μg/mL), LPS + BCP (10 μg/mL), LPS + BCP +AM630 (100 nM), when it reached confluence. In particular, LPS, LPS + BCP and LPS + BCP + AM360 were tested in a 96-well plate at a density of 8 × 10^4^ cells/well for 24 h to evaluate the cytotoxic effect. The tetrazolium dye MTT 3-(4,5-dimethylthiazol-2-yl)-2,5-diphenyltetrazolium bromide (Sigma Aldrich, Milan, Italy) was dissolved in sterile filtered PBS, and 20 μL of the mixture were added into each well 5 h before the end of the 24 h of incubation. Medium was removed and the insoluble formazan crystals were dissolved with dimethyl sulfoxide (DMSO; 200 μL/well) following 5 h. The difference between the values obtained at 540 and 620 nm of absorbance was used to calculate the average of replicates and to evaluate cytotoxicity. Results were expressed as % of cell viability compared to untreated cells and reported as means and SD.

### 2.4. Measurements of Cytokines by Enzyme-Linked Immunosorbent Assay (ELISA)

TNF-α, IL-1β, IL-13, IL-6 and IL-17A were measured in the cell supernatants. The cytokines under investigation were evaluated using Enzyme-Linked Immunosorbent Assay (ELISA) kits (Abcam, Cambridge, UK) in agreement with the instructions reported by the manufacturer. All the samples were evaluated in duplicate and the obtained results were interpolated with the pertinent standard curves. To evaluate the sample, the means of the duplicated sample were used and expressed in pg/mL [19,20].

### 2.5. Real Time Quantitative PCR Amplification (RTqPCR)

Total RNA was extracted from GF and EC cells for RTqPCR using Trizol LS Reagent (Invitrogen, Carlsbad, CA, USA). Two micrograms of total RNA was reverse transcribed in a final volume of 20 μL using a Superscript VILO kit (Invitrogen). cDNA (1 μL) was added to the EvaGreen qPCR Master Mix (Biotium Inc., Fremont, CA, USA) (20 μL per well). The final primer concentration selected to perform the analysis was 10 μM. Samples were run in duplicate and β-actin was used as an endogenous control. Results were calculated using the 2^−ΔΔCT^ method and expressed as n-fold increase in gene expression using the CTRL group as the calibrator [21]. Primers used for targets and reference genes are listed in Table 1.

### 2.6. Statistical Analysis

Results were statistically analyzed calculating standard deviation (SD). Data are expressed as the mean ± SD and the values reported are the results of at least five experiments performed in duplicate. All assays were repeated three times to ensure reproducibility. The different groups were compared and analyzed using one-way ANOVA with a Tukey post-test for comparison between the different groups. A *p* value < 0.05 was considered significant. Graphs were prepared using GraphPad Prism (version 5.0 for Windows, San Diego, CA, USA).

## 3. Results

### 3.1. BCP Reverts the Inflammatory Phenotype Induced by LPS in Gingival Fibroblasts and Oral Mucosa Epithelial Cells

**Figure 1:**
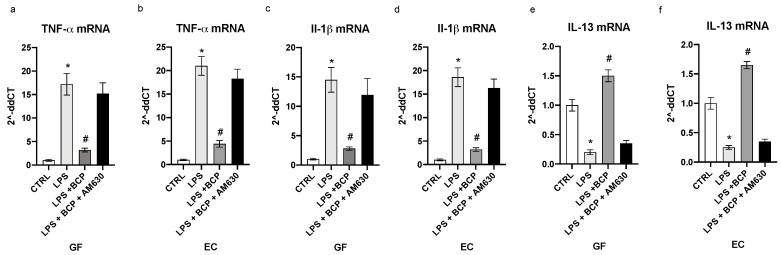
The graphs represent qPCR results of TNF-α (**a**), IL-1β (**c**), IL-13 (**e**) mRNA expression from GF cells and TNF-α (**b**), IL-1β (**d**), IL-13 (**f**) mRNA expression from EC cells. Values are expressed as the means and SD. * *p* < 0.0001 vs. CTRL; # *p* < 0.0001 vs. LPS.

**Figure 2:**
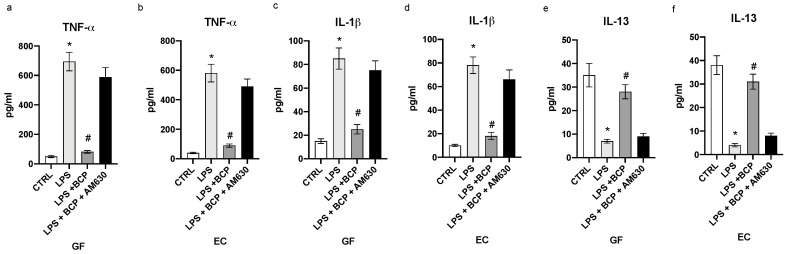
The graphs represent the levels of TNF-α (**a**), IL-1β (**c**) and IL-10 (**e**) in cell supernatants from GF cells and TNF-α (**b**), IL-1β (**d**), IL-13 (**f**) levels in cell supernatants from EC cells. Levels of cytokines were evaluated by immunosorbent assay (ELISA). Values are expressed as the means and SD. * *p* < 0.0001 vs. CTRL; # *p* < 0.0001 vs. LPS.

LPS challenge resulted in a marked expression of TNF-α and IL-1β with a concomitant reduced mRNA of IL-13 in both gingival fibroblasts and oral mucosa epithelial cells (*p* < 0.0001 vs. CTRL; Figure 1). To confirm the full induction of the inflammatory phenotype, we measured the mature proteins in the cell supernatants. TNF-α and IL-1β were markedly increased, while IL-13 significantly diminished in the supernatants of GF and EC cells (*p* < 0.0001 vs. CTRL; Figure 2). BCP incubation in LPS stimulated GF and EC cells suppressed the increased mRNA for the inflammatory cytokines TNF-α and IL-1β and caused a marked enhancement in the expression of the message of the anti-inflammatory cytokine IL-13 (*p* < 0.0001 vs. LPS; Figure 1). Overlapping results were observed when mature proteins were used as readouts (*p* < 0.0001 vs. LPS; Figure 2). To dissect out the role of the CB2 receptors in the effect of BCP, we performed experiments in which a specific antagonist of this receptor subtype (AM630) was added in cell culture 2 h before BCP. Blockade of the CB2 receptor reverted the positive effect of the biomolecule on the inflammatory phenotype (Figure 1 and Figure 2).

### 3.2. BCP Modulates Upstream Signals that Trigger the First Phase of the Inflammatory Response

**Figure 3:**
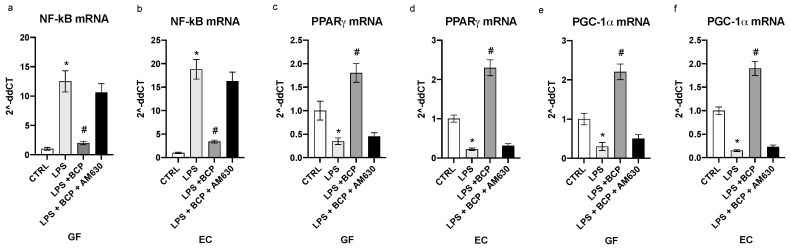
The graphs represent the qPCR results of NF-kB (**a**), PPARγ (**c**), PGC1α (**e**) mRNA expression from GF cells and NF-kB (**b**), PPARγ (**d**), PGC1α (**f**) mRNA expression from EC cells. Values are expressed as the means and SD. * *p* < 0.0001 vs. CTRL; # *p* < 0.0001 vs. LPS.

The transcription factor NF-κB was markedly induced by the LPS challenge in both human gingival fibroblasts and oral mucosa epithelial cells (*p* < 0.0001 vs. CTRL; Figure 3a,b). BCP treatment in GF and EC cells suppressed the mRNA for the transcription factor and this effect was abrogated by AM630, a specific antagonist of CB2 receptors (*p* < 0.0001 vs. LPS; Figure 3a,b). LPS also produced a diminished PPARγ and PGC-1α expression in gingival fibroblasts (*p* < 0.0001vs. CTRL; Figure 3c,e) and epithelial cells (*p* < 0.0001 vs CTRL; Figure 3d,f). BCP treatment prompted a marked enhancement in the expression of both the nuclear receptor and its co-activator when compared to cell cultures challenged with LPS alone (*p* < 0.0001 vs. LPS; Figure 3c–f). The antagonist of the CB2 receptor AM630 reverted the effects of BCP in GF and EC cells (Figure 3).

### 3.3. BCP Halts the Second Phase of the Inflammatory Response in Gingival Fibroblasts and Oral Mucosa Epithelial Cells

**Figure 4:**
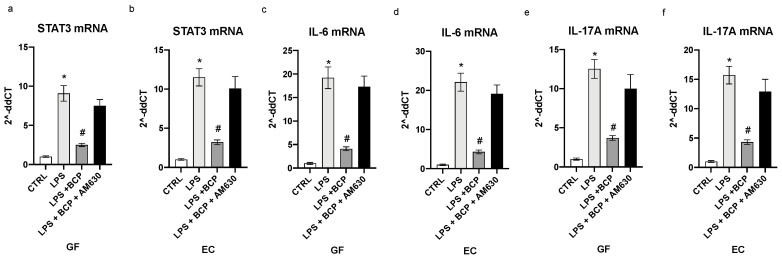
The graphs represent qPCR results of STAT3 (**a**), IL-6 (**c**), IL-17A (**e**) mRNA expression from GF cells and STAT3 (**b**), IL-6 (**d**), IL-17A (**f**) mRNA expression from EC cells. Values are expressed as the means and SD. * *p* < 0.0001 vs. CTRL; # *p* < 0.0001 vs. LPS.

**Figure 5:**
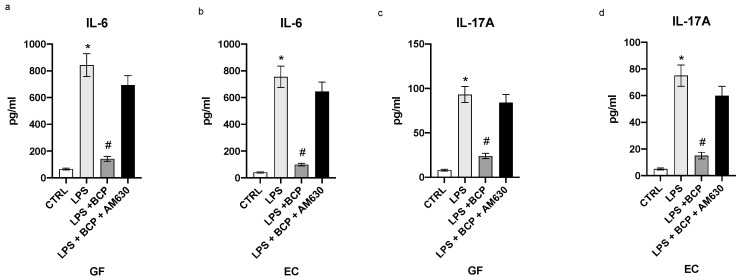
The graphs represent the levels of IL-6 (**a**), IL-17A (**c**) in cell supernatants from GF cells and IL-6 (**b**), IL-17A (**d**) levels in cell supernatants from EC cells. Levels of cytokines were evaluated by immunosorbent assay (ELISA). Values are expressed as the means and SD. * *p* < 0.0001 vs. CTRL; # *p* < 0.0001 vs. LPS.

STAT-3 serves as a pathophysiological connection between the first and the second phase of the acute inflammatory response. LPS stimulation prompted a robust increase in the message for this upstream signal in both gingival fibroblasts and oral mucosa epithelial cells (*p* < 0.0001 Figure 4a,b). BCP incubation markedly dampened STAT-3 expression in both GF and EC cells and CB2 receptors blockade by AM630 cancelled the BCP effects (*p* < 0.0001 Figure 4a,b). IL-6 is released during the acute phase of the inflammatory reaction and it is the cytokine that promotes the transition from the first phase to the second wave of the acute inflammatory cascade. This step, if it is not adequately modulated, results in a chronic inflammatory condition. BCP was able to decrease the IL-6 message and mature protein, both markedly stimulated by LPS challenge in GF an EC cells (*p* < 0.0001 Figure 4c,d; Figure 5a,b). Again, the pharmacological blockade of the CB2 receptor brought about by AM630 reverted the BCP effects (*p* < 0.0001 Figure 4c,d; Figure 5a,b). Dysregulated activation of STAT-3 promotes an aberrant production of IL-17A, a cytokine that orchestrates the accumulation of inflammatory cells as neutrophils to the inflammatory scene. If the release is persistent, it may cause a negative remodeling of the inflamed tissue, thus sustaining and maintaining a chronic inflammation scenario. LPS challenge caused an elevation in both IL-17A mRNA and mature protein in both GF and EC cells (*p* < 0.0001 Figure 4c,f; Figure 5b,d). BCP incubation markedly reduced IL-17A expression and this effect was abolished by AM630, a cannabinoid CB2 receptor antagonist (*p* < 0.001 Figure 4e,f; Figure 5c,d). This experiment indicates that BCP efficiently interrupts the transition towards chronic inflammation.

### 3.4. BCP Does Not Affect Cell Viability

**Figure 6:**
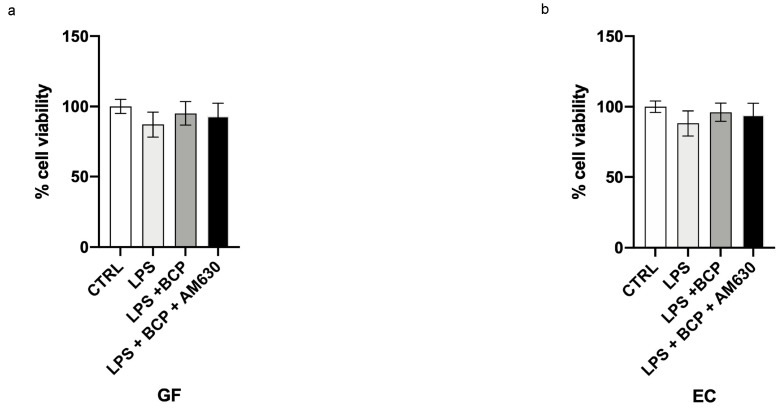
The graphs show the cytotoxicity assay at 24 h in GF cells (**a**) and EC cells (**b**). Values are expressed as the means and SD.

One hundred percent of viability was observed on control cells following 24 h. The incubation with β-caryophyllene did not affect GF and EC viability, thus demonstrating that this natural product does not have a cytotoxic effect and does not affect cell viability. Furthermore LPS incubation did not change cell viability (Figure 6a,b).

## 4. Discussion

In the present study we reproduced “in vitro” an experimental paradigm to mimic oral mucositis. To this aim we primed with LPS human gingival fibroblasts and oral mucosa epithelial cells, two main components of the periodontium. Both cell types acquired an inflammatory phenotype characterized by a sustained inflammatory cascade that was orchestrated by the transcription factors NF-κB and STAT3. Indeed, our experimental model is a slight modification of a previous published bioassay for oral mucositis [15]. However, we used LPS to trigger the inflammatory phenotype instead of recombinant cytokines, as in the previously published model [22].

LPS stimulation may have some theoretical advantages over cytokine stimulation: it may reproduce more closely the clinical scenario where infective stimuli and micro-organisms, at least in peri-implantitis, play an important role and may activate more efficiently the intracellular upstream signals that regulate and coordinate the inflammatory storm. In agreement with this reasoning it has been shown that in oral mucositis, pathogens are main participants in the triggering of the inflammatory cascade by engaging pathogen associated molecular patterns (PAMPs), such as the toll-like receptor that is targeted by LPS [23].

Oral mucositis management is theoretically easy to accomplish, but practically hard to implement in the clinical setting. Several therapeutics have been proposed for the treatment of oral mucositis: they include local antiseptic and cytoprotective agents, laser therapy and cryotherapy as well as pharmacological approaches such as anti-inflammatory drugs, recombinant cytokines and growth factors. Among these curative strategies, keratinocyte growth factor-1 is the only medicine that has received approval by the U.S. Food and Drug administration and by the European Medicine Agency for the management of oral mucositis, but its use is restricted to “at-high-risk” population [24,25]. Phyto-therapy has been suggested as a rationale strategy in the management of oral mucositis [26,27,28]. In this context BCP may represent an interesting molecule. It is a bicyclic sesquiterpene obtained by extraction from copaiba (Copaifera spp) and marijuana/hemp (Cannabis spp) that has gained the Food Drug Administration (FDA) authorization in light of its interesting curative profile. The compound has a long history of use in complementary therapy because of its ability to reduce inflammation and to cause analgesia [29]. β-caryophyllene engages with high affinity the cannabinoid CB2 receptors which are mainly localized in the immune system and immune-derived cells [30]. Since BCP does not bind the type 1 cannabinoid receptors, it is devoid of action at the Central Nervous System.

Several targets have been proposed to design a curative approach for oral mucositis, but so far, no attempt has been carried out to explore the feasibility of positively modulating the CB2 receptor. Our results suggest that BCP succeeded in turning off the inflammatory phenotype of GF and EC cells that is the main pathophysiological hallmark of oral mucositis [31]. BCP reduced relevant inflammatory cytokines such as TNF-α and IL-1β and augmented the expression of the anti-inflammatory IL-13. The positive effects brought about by BCP were abrogated by a specific antagonist of the CB2 receptor, thus clearly pointing out that this specific receptor subtype represents the mode of action of this natural compound. Furthermore, the results unmask for the first time that the CB2 receptor has potential for becoming a target candidate to allow an innovative drug design for medicines useful in the management of oral mucositis. We also explored the first steps of the intracellular biomolecular pathway that is modulated by the blockade of the CB2 receptor and we identified that the transcription factor NF-κB is the upstream molecular signal that is targeted by this therapeutic strategy. This result is of paramount importance: in fact, the transcription factor has been recognized as being one of the main actors in the initiation of oral mucositis and therefore the strategy of the CB2 blockade is able to intercept the very early event in the boosting of the inflammatory cascade. PPARγ is a nuclear receptor that may be considered as an “atypical transcription factor” able to activate anti-inflammatory signals. Indeed, pharmacological stimulation of PPARγ exerts beneficial activity in oral mucositis [13,14]. In agreement with these findings we also investigated PPARγ and its co-activator PGC-1α and we found that the expression of this intracellular signal was “downregulated” in our model of oral mucositis. Interestingly stimulation of the CB2 receptor by BCP also caused an upregulation of this protective intracellular signal, thus confirming previous studies suggesting the occurrence of a cross-talk between CB2 and PPAR-γ receptors [32].

Inflammation is a complex cascade of events that may lead to its resolution or, alternatively, to its stalling into a chronic condition that renders the management of the clinical condition hard to accomplish. The molecular events involved in this scenario are: an exaggerated production of IL-6 [33] and the consequent activation of the transcription factor STAT3 [34] that primes the production and release of several additional cytokines that sustain and maintain the inflammatory state. Among those cytokines, IL-17A is of paramount importance in the context of oral disease. Elevated levels of IL-17A have been measured in periodontal diseases characterized by high grade chronic inflammation [35,36]. In this clinical setting IL-17A orchestrates a coordinated recruitment of inflammatory cells that amplifies the maladaptive mechanisms underlying the maintenance of a persistent inflammation. Interestingly, BCP succeeded in counteracting this second wave of the host inflammatory response.

All these data, taken together highlight the great therapeutic potential of BCP for oral mucositis; in fact it possesses a dual mechanism of action; inhibition of NF-κB and activation of PPAR-γ that amplifies its efficacy in the first phase of inflammation and later it halts, by inhibiting STAT3, the second phase of the inflammatory response. Finally, it has been shown that BCP has an anti-cancer effect [37] that may enhance the appropriateness of this treatment, at least for oral mucositis due to chemotherapy or radiotherapy.

In conclusion, our data show for the first time that BCP has a marked efficacy in a preclinical in vitro model of oral mucositis: this effect, in light of its high translational potential, deserves to be confirmed in a clinical setting.

## Figures and Tables

**Table 1:** Primer list.

Gene	Sequence
*β-actin*	Fw:5′AGAGCTACGAGCTGCCTGAC3′
	Rw:5′AGCACTGTGTTGGCGTACAG3′
*TNF-α*	Fw:5′CAGAGGGCCTGTACCTCATC3′
	Rw:5′GGAAGACCCCTCCCAGATAG3′
*IL-1β*	Fw:5′TGAGCTCGCCAGTGAAATGA3′
	Rw:5′AGATTCGTAGCTGGATGCCG3′
*IL-13*	Fw:5′CATGGCGCTTTTGTTGACCA 3′
	Rw:5′AGCTGTCAGGTTGATGCTCC3′
*NF-κB*	Fw:5′CCTGGATGACTCTTGGGAAA3′
	Rw:5′TCAGCCAGCTGTTTCATGTC3′
*PPAR-γ*	Fw:5′TCGACCAGCTGAATCCAGAG3′
	Rw:5′GGGGGTGATGTGTTTGAACTTG3′
*PGC-1α*	Fw:5′CATGTGCAACCAGGACTCTGA3′
	Rw:5 GCGCATCAAATGAGGGCAAT3′
*STAT3*	Fw:5′GAGCTGCACCTGATCACCTT3′
	Rw:5′CCCAGAAGGAGAAGCCCTTG3′
*IL-6*	Fw:5′TTCGGTCCAGTTGCCTTCTC3′
	Rw:5′CAGCTCTGGCTTGTTCCTCA3′
*IL-17A*	Fw:5′CTGTCCCCATCCAGCAAGAG3′
	Rw:5′AGGCCACATGGTGGACAATC3′

## References

[1] Elting L.S., Cooksley C.D., Chambers M.S., Garden A.S. (2007). Risk, Outcomes, and Costs of Radiation-Induced Oral Mucositis Among Patients With Head-and-Neck Malignancies. Int. J. Radiat. Oncol. Biol. Phys..

[2] Elting L.S., Keefe D.M., Sonis S.T., Garden A.S., Spijkervet F.K., Barasch A., Tishler R.B., Canty T.P., Kudrimoti M.K., Vera-Llonch M. (2008). Patient-reported measurements of oral mucositis in head and neck cancer patients treated with radiotherapy with or without chemotherapy. Cancer.

[3] Murphy B.A., Beaumont J.L., Isitt J., Garden A.S., Gwede C.K., Trotti A.M., Meredith R.F., Epstein J.B., Le Q.T., Brizel D.M. (2009). Mucositis-Related Morbidity and Resource Utilization in Head and Neck Cancer Patients Receiving Radiation Therapy With or Without Chemotherapy. J. Pain Symptom Manag..

[4] Lalla R.V., Sonis S.T., Peterson D.E. (2008). Management of oral mucositis in patients who have cancer. Dent. Clin. N. Am..

[5] Pjetursson B.E., Asgeirsson A.G., Zwahlen M., Sailer I. (2014). Improvements in implant dentistry over the last decade: Comparison of survival and complication rates in older and newer publications. Int. J. Oral Maxillofac. Implants.

[6] Albrektsson T., Donos N. (2012). Implant survival and complications. The third EAO consensus conference 2012. Clin. Oral Implants Res..

[7] Atieh M.A., Alsabeeha N.H.M., Faggion C.M., Druncan W.J. (2013). The frequency of peri-implant diseases: A systematic review and metaanalysis. J. Periodontol..

[8] Abrahamsson I., Berglundh T. (2009). Effects of different implant surfaces and designs on marginal bone-level alterations: A review. Clin. Oral Implants Res..

[9] Sonis S.T. (2010). New thoughts on the initiation of mucositis. Oral Dis..

[10] Mombelli A., Oosten M.A.C., Schürch E., Land N.P. (1987). The microbiota associated with successful or failing osseointegrated titanium implants. Oral Microbiol. Immunol..

[11] Sardaro N., Della Vella F., Incalza M.A., Di Stasio D., Lucchese A., Contaldo M., Laudadio C., Petruzzi M. (2019). Oxidative Stress and Oral Mucosal Diseases: An Overview. In Vivo.

[12] Curra M., Pellicioli A.C., Filho N.A., Ochs G., Matte U., Filho M.S., Martins M.A., Martins M.D. (2015). Photobiomodulation reduces oral mucositis by modulating NF-kB. J. Biomed. Opt..

[13] Mangoni M., Sottili M., Gerini C., Desideri I., Bastida C., Pallotta S., Castiglione F., Bonomo P., Meattini I., Greto D. (2017). A PPAR gamma agonist protects against oral mucositis induced by irradiation in a murine model. Oral Oncol..

[14] Sottili M., Mangoni M., Gerini C., Salvatore G., Castiglione F., Desideri I., Bonomo P., Meattini I., Greto D., Loi M. (2018). Peroxisome proliferator activated receptor gamma stimulation for prevention of 5-fluorouracil-induced oral mucositis in mice. Head Neck.

[15] Panahipour L., Nasserzare S., Amer Z., Brücke F., Stähli A., Kreissl A., Haiden N., Gruber R. (2019). The anti-inflammatory effect of milk and dairy products on periodontal cells: An in vitro approach. Clin. Oral Investig..

[16] Russo E.B. (2016). Beyond Cannabis: Plants and the Endocannabinoid System. Trends Pharmacol. Sci..

[17] Sharma C., Al Kaabi J.M., Nurulain S.M., Goyal S.N., Kamal M.A., Ojha S. (2016). Polypharmacological Properties and Therapeutic Potential of β-Caryophyllene: A Dietary Phytocannabinoid of Pharmaceutical Promise. Curr. Pharm. Des..

[18] D’Ascola A., Irrera N., Ettari R., Bitto A., Pallio G., Mannino F., Atteritano M., Campo G.M., Minutoli L., Arcoraci V. (2019). Exploiting curcumin synergy with natural products using quantitative analysis of doe-effect relationships in an in vitro model of osteoarthritis. Front. Pharmacol..

[19] Marini H., Polito F., Altavilla D., Irrera N., Minutoli L., Calò M., Adamo E.B., Vaccaro M., Squadrito F., Bitto A. (2010). Genistein aglycone improves skin repair in an incisional model of wound healing: A comparison with raloxifene and oestradiol in ovariectomized rats. Br. J. Pharmacol..

[20] Minutoli L., Marini H., Rinaldi M., Bitto A., Irrera N., Pizzino G., Pallio G., Calò M., Adamo E.B., Trichilo V. (2015). A dual inhibitor of cyclooxygenase and 5-lipoxygenase protects against kainic acid-induced brain injury. Neuromolecular Med..

[21] Pizzino G., Irrera N., Bitto A., Pallio G., Mannino F., Arcoraci V., Aliquò F., Minutoli L., De Ponte C., D’Andrea P. (2017). Cadmium-Induced Oxidative Stress Impairs Glycemic Control in Adolescents. Oxid. Med. Cell Longev..

[22] Irrera N., D’Ascola A., Pallio G., Bitto A., Mazzon E., Mannino F., Squadrito V., Arcoraci V., Minutoli L., Campo G.M. (2019). β-Caryophyllene Mitigates Collagen Antibody Induced Arthritis (CAIA) in Mice Through a Cross-Talk between CB2 and PPAR-γ Receptors. Biomolecules.

[23] Tang D., Kang R., Coyne C.B., Zeh H.J., Lotze M.T. (2012). PAMPs and DAMPs: Signal 0s that spur autophagy and immunity. Immunol. Rev..

[24] Hou J., Zheng H., Li P., Liu H., Zhou H., Yang X. (2018). Distinct shifts in the oral microbiota are associated with the progression and aggravation of mucositis during radiotherapy. Radiother. Oncol..

[25] Lalla R.V., Bowen J., Barasch A., Elting L., Epstein J., Keefe D.M., McGuire D.B., Migliorati C., Nicolatou-Galitis O., Peterson D.E. (2014). Mucositis Guidelines Leadership Group of the Multinational Association of Supportive Care in Cancer and International Society of Oral Oncology (MASCC/ISOO). MASCC/ISOO clinical practice guidelines for the management of mucositis secondary to cancer therapy. Cancer.

[26] Li C.L., Huang H.L., Wang W.C., Hua H. (2016). Efficacy and safety of topical herbal medicine treatment on recurrent aphthous stomatitis: A systemic review. Drug Des. Devel. Ther..

[27] Zhang Y., Ng K.H., Kuo C.Y., Wu D.J. (2018). Chinese herbal medicine for recurrent aphthous stomatitis: A protocol for systematic review and meta-analysis. Medicine.

[28] Baharvand M., Jafari S., Mortazavi H. (2017). Herbs in Oral Mucositis. J. Clin. Diagn. Res..

[29] La Porta C., Bura S.A., Llorente-Onaindia J., Pastor A., Navarrete F., García-Gutiérrez M.S., De la Torre R., Manzanares J., Monfort J., Maldonado R. (2015). Role of the endocannabinoid system in the emotional manifestations of osteoarthritis pain. Pain.

[30] Machado K.D.C., Islam M.T., Ali E.S., Rouf R., Uddin S.J., Dev S., Shilpi J.A., Shill M.C., Reza H.M., Das A.K. (2018). A systematic review on the neuroprotective perspectives of beta-caryophyllene. Phytother. Res..

[31] Sonis S.T. (2007). Pathobiology of oral mucositis: Novel insights and opportunities. J. Support. Oncol..

[32] Youssef D.A., El-Fayoumi H.M., Mahmoud M.F. (2019). Beta-caryophyllene protects against diet-induced dyslipidemia and vascular inflammation in rats: Involvement of CB2 and PPAR-γ receptors. Chem. Biol. Interact..

[33] Hernández-Caldera A., Vernal R., Paredes R., Veloso-Matta P., Astorga J., Hernández M. (2018). Human periodontal ligament fibroblasts synthesize C-reactive protein and Th-related cytokines in response to interleukin (IL)-6 trans-signalling. Int. Endod. J..

[34] Bharadwaj U., Kasembeli M.M., Robinson P., Tweardy D.J. (2020). Targeting Janus Kinases and Signal Transducer and Activator of Transcription 3 to Treat Inflammation, Fibrosis, and Cancer: Rationale, Progress, and Caution. Pharmacol. Rev..

[35] Vernal R., Dutzan N., Chaparro A., Puente J., Antonieta Valenzuela M., Gamonal J. (2005). Levels of interleukin-17 in gingival crevicular fluid and in supernatants of cellular cultures of gingival tissue from patients with chronic periodontitis. J. Clin. Periodontol..

[36] Mitani A., Niedbala W., Fujimura T., Mogi M., Miyamae S., Higuchi N., Abe A., Hishikawa T., Mizutani M., Ishihara Y. (2015). Increased expression of interleukin (IL)-35 and IL-17, but not IL-27, in gingival tissues with chronic periodontitis. J. Periodontol..

[37] Irrera N., D’Ascola A., Pallio G., Bitto A., Mannino F., Arcoraci V., Rottura M., Ieni A., Minutoli L., Metro D. (2020). β-Caryophyllene Inhibits Cell Proliferation through a Direct Modulation of CB2 Receptors in Glioblastoma Cells. Cancers.

